# Impact of the COVID-19 pandemic on HPV vaccine uptake in a predominantly Hispanic Border Community: A retrospective cross-sectional analysis of the "Tiempo de Vacunarte Program"

**DOI:** 10.1186/s13690-024-01318-0

**Published:** 2024-06-24

**Authors:** Phong Nguyen, Jessica Calderon-Mora, Vishwajeet Singh, Amir Hernandez, Sonya Roy, Jennifer Molokwu

**Affiliations:** 1 Department of Family and Community Medicine, Texas Tech University Health Sciences Center El Paso, 9849 Kenworthy Street, El Paso, TX 79924 USA; 2 Office of Research, Biostatistics and Epidemiology Consulting Lab El Paso, Texas Tech University Health Sciences Center El Paso, El Paso, TX USA; 3 Paul L. Foster School of Medicine Texas, Tech University Health Sciences Center El Paso, El Paso, TX USA; 4 Department of Population Health, The University of Texas at Austin Dell Medical School, Austin, TX USA

**Keywords:** Hispanic, Outcomes, HPV, Vaccination, COVID-19, Pandemic

## Abstract

**Background:**

Human Papillomavirus (HPV) is implicated in the pathogenesis of cancer in the cervix, vagina, throat and anogenital region. Although HPV vaccination rates in the Hispanic community have increased owing to public health efforts, the COVID-19 pandemic has brought unique public health challenges and contributed to health inequity in this population.

**Methods:**

To evaluate the impact of the COVID-19 pandemic on HPV vaccine uptake in a program designed to improve HPV vaccination rate in a predominantly Hispanic community in the border region of Texas (Tiempo de Vacunarte [time to get vaccinated]), we performed a retrospective cross-sectional analysis to evaluate the uptake of the first dose of HPV vaccine series among eligible adolescents and adults before (2016–2019), during (2020–2021), and after the COVID-19 pandemic (2022–2023).

**Results:**

We observed a decrease in HPV vaccine uptake during the pandemic (69.59% vs. 89.92%) and post-pandemic (76% vs. 89.92%) compared to the pre-pandemic period. After adjusting for confounding factors, the reduction in the odds ratio was more pronounced in the pandemic (OR = 0.091, *p* < 0.001) and post-pandemic (OR = 0.109, *p* < 0.001) periods.

**Conclusion:**

Our findings suggest that the COVID-19 pandemic significantly impacted the uptake of the HPV vaccine in a comprehensive intervention program to increase HPV vaccination in a border community.

**Table:** 

Text box 1. Contributions to the literature
• By quantifying the decline in HPV vaccine uptake in an evidence-based, culturally tailored, multi-component program during and after the COVID-19 pandemic, the study provides concrete evidence of the pandemic's negative impact on public health initiatives.
• The study highlights the need for resilient public health strategies that can withstand such disruptions.
• Policy Implications: The findings suggest that additional support and targeted efforts may be needed to restore and improve vaccination rates post-pandemic.

## Introduction

Human papillomavirus (HPV) is the most common sexually transmitted virus in the United States [42] and is classified into low-risk and high-risk types. Low-risk types can cause benign conditions, while high-risk types are implicated in the causal pathways of cancers of the vulva, vagina, cervix, penis, throat, and anogenital regions [17]. Despite a decline in cervical cancer rates in the United States, health disparities persist and disproportionately affect the Hispanic population, especially among Hispanic communities in South Texas [27]. Morales-Campos and colleagues reported higher rates of cervical cancer incidence (11 vs. 9 per 100,000) and mortality (3 vs. 2 per 100,000) among individuals of Hispanic origin from South Texas compared to other parts of the country [29]. A similar trend was observed for penile cancer, where males of Hispanic origin had higher rates (1.3 vs. 0.8 per 100,000) than males of non-Hispanic origin [13]. These statistics underscore the urgency of addressing health disparities, especially given the lower HPV vaccine completion rate among people of Hispanic origin in Texas compared to the national average (45% vs. 54.5%) [29]

HPV vaccines are highly effective in the prevention of cervical cancer. Between 2008 and 2014, the percentage of cervical precancers caused by HPV strains 16 and 18 decreased from 55.2% to 33.3% in vaccinated women [25]. In another study from 2006 to 2017 that examined HPV vaccine efficacy among nearly 1.7 million women in Sweden, there was close to a 90% reduction in the incidence rate of cervical cancer among women who were vaccinated compared with those who had not been vaccinated [21]. A recent randomized clinical trial, the KEN SHE study, strengthened evidence that a single-dose HPV vaccine is equally effective as a multi-dose HPV vaccine in reducing the incidence of oncogenic HPV infection in women over an 18-month study time frame between December 2018 and June 2021 [4]. HPV vaccine has also been shown to be effective in males; McClung and colleagues reported a 65% reduction in external genital lesions in males who received the HPV vaccine [25]. Despite the widely documented evidence for the efficacy and safety of the HPV vaccine, there remains a disparity in the uptake and completion of the HPV vaccine series among adolescents compared to other vaccines recommended for the same age [18].

A systematic review by Rambout and colleagues identified cost as the most common self-reported barrier to HPV vaccine access [37]. Other studies have identified systemic and practical barriers to HPV vaccine access, including lack of knowledge, inadequate healthcare provider recommendations, lack of insurance, difficulty obtaining transportation, problems with booking appointments, and the need for additional office visits [37, 39]. *Several barriers have been noted as specific among Hispanics, including fear of potentially promoting early sexual activity, fear of adverse reactions, and health misinformation *[15, 20]*.* De and Budhwani reported the persistence of health disparities among African Americans and other minority groups, excluding Asian Americans and foreign-born individuals residing in the United States [10]. In addition, there are self-reported factors that contribute to the reluctance to obtain the HPV vaccine, including concerns about vaccine safety and side effects, perceived low susceptibility to HPV infection, perceived low access to the vaccine, societal norms, religious background, perception that the vaccine is unnecessary, and fear of needles [37].

Compounding these existing barriers, the COVID-19 pandemic has posed new challenges to vaccine uptake. A study estimated that 7.9% of children/adolescents had a COVID-related missed visit, with a higher percentage of these children/adolescents being from racial and ethnic minority groups, living below the poverty level, having a mother without a college degree, and living in the western United States [3]. Similarly, Moya and colleagues found that 43.5% of participants in El Paso reported that the pandemic impacted their access to health and human services; the HPV vaccine was considered unnecessary since COVID-19 had brought more pressing concerns [31]. There is also evidence that increasing vaccine hesitancy around the pandemic has played a role in the decrease in HPV vaccine uptake. One study showed that 11% of pediatricians reported increased HPV vaccine hesitancy since the beginning of the COVID-19 pandemic [41].

Our study aimed to evaluate the effects of the pandemic on the uptake of one dose of the HPV vaccine in a comprehensive multi-component HPV vaccine intervention program called Tiempo de Vacunarte, which was designed to improve the HPV vaccination rate in El Paso, a predominantly Hispanic community in the border region of Texas. The program consisted of multiple interventions, including education, navigation to resources, and provision of no-cost vaccines to the public and El Paso community members who met the eligibility criteria. The program had previously been found to improve immunization rates, HPV knowledge, HPV awareness, and the intention to vaccinate in the border population [28]. However, the COVID-19 pandemic has brought unique challenges to the program. Thus, we sought to quantify the impact of the pandemic on HPV vaccine uptake in this program.

## Methods

### Study design

We conducted a retrospective cross-sectional analysis, assessing HPV vaccine uptake before (2016–2019), during (2020–2021), and after (2022–2023) the COVID-19 pandemic, utilizing two cohorts: Tiempo 1 (2016–2019) and Tiempo 2 (2020–2022).

### Study setting and recruitment

El Paso is a predominantly Hispanic county on the United States-Mexico border region in Texas, with a population of 867,947 [33]. This county contends higher poverty rates and poorer health coverage than the national average [36]. *Tiempo de Vacunarte is a grant-funded program established in El Paso County in 2016. It is an evidence-based, culturally tailored, multi-component program to reduce the burden of HPV-associated cancers by improving HPV vaccine knowledge and increasing vaccine uptake. The program provides no-cost vaccines for community members who are uninsured or underinsured. In contrast, for those with insurance coverage, the program provides health education and navigation to their primary care provider.* Participants in the program were primarily community-based and recruited from multiple community sites with the aid of community health workers. Sites included, but were not limited to, community centers, health fairs, community colleges, trade schools, school districts, churches, and food banks.

### Eligibility

The eligibility criteria for the program were age, insurance status, and possession of a Texas address per the requirement of the grant-funding agency. For Tiempo 1, the age requirements were adults aged 18–26 or parents/guardians of children aged 9–17; for Tiempo 2, the adult age requirement was expanded to include shared decision-making for individuals aged 27–45 who had not completed the vaccine schedule, were uninsured/underinsured, and had Texas residences. The age criteria were determined using the Advisory Committee of Immunization Practices recommendations for HPV vaccination [24].

### Intervention

The program sought individuals with access to care barriers. The intervention consisted of outreach, education, navigation, and providing no-cost vaccines to eligible individuals. The development of the educational material for the program was guided by the Health Belief Model (HBM) and informed by the findings of focus groups previously conducted in the community [34]. The focus groups identified cultural concerns, barriers, and knowledge gaps specific to this community, and this informed our adaptation of the available material on the Centers for Disease Control and Prevention's website [32]. The educational materials were culturally tailored to the community and were available in Spanish and English. The sessions were delivered by bilingual community health workers, also known as promotoras, to further address cultural and language barriers. The navigation component was delivered by program navigators, who helped participants with community resources, scheduling, and transportation assistance. Access to the vaccine was provided after eligibility criteria were met and education was delivered. For those eligible for the no-cost vaccine, a certified medical assistant provided immunization at the recruitment site or scheduled a vaccine administration appointment at either the participant's home or a collaborating community site. *Those who were insured were referred to their providers for vaccination.* If a participant reported receiving an HPV vaccine outside the program during the follow-up period, this information was verified using the state immunization registry. Details on the development and implementation of Tiempo have been previously published [28].

During the height of the pandemic, specific adaptations were made, which included outreach and education via telephone and other audiovisual methods such as Zoom and Webex, as well as providing vouchers for participants to receive their vaccines at their local Walgreens or Immunize El Paso office. As the community returned to in-person activities, we transitioned to in-person outreach and education. However, we maintained the option for individuals to receive a voucher for vaccination if they prefer not to be vaccinated onsite or return to our office for follow-up vaccination.

### Outcome

*The primary outcome measure was HPV vaccine uptake. Individuals were contacted in person or virtually and received information about the program(vaccine encounters). They were recruited if they met the criteria and agreed to receive services through our program. We maintained records of recruited participants on a HIPAA-compliant program database. We documented whether they went on to receive at least one vaccine dose, which we defined as vaccine uptake/initiation.*

### Covariates

*We obtained demographic information such as age, sex, level of education, race, ethnicity, country of birth, household income, and length of residence in the United States. Previous work identified these covariates as impacting HPV vaccine completion in our community* [28]*. Our statistical analysis did not include the country of birth and length of residence in the United States due to a lot of missing data in this variable.*

### Statistical analysis

Categorical variables were presented as numbers and percentages, whereas quantitative variables were presented as means, standard deviations, medians, and interquartile ranges. We used the chi-square or Fisher's exact test to explore the association between categorical variables. Similarly, we used a t-test or Wilcoxon rank-sum test to compare the quantitative variables between the two groups. When there were more than two groups, we performed a one-way analysis of variance or Kruskal–Wallis one-way analysis of variance. Finally, we performed unadjusted and adjusted logistic regression analysis to determine the association between HPV vaccination uptake/initiation with the periods before (2016–2019), during (2021–2021), and after the COVID-19 pandemic (2022–2023). In our statistical analysis, we combined the data for vaccine uptake across the years. The results were presented using odds ratio (OR) and corresponding 95% confidence intervals (95% CI), with statistical significance set at *p* < 0.05. All analyses were performed using STATA statistical software (version 17).

## Results

This study examined HPV vaccine initiation rates among program participants during three distinct periods: pre-pandemic (before 2020), during the pandemic (2020 to 2021), and post-pandemic (2022 to 2023).

### Demographic characteristics

A majority of our participants (96.86%) were of Hispanic origin*.* The mean age in years of participants was significantly higher during the pandemic (30.4 vs. 18, *p* < 0.001) and post-pandemic (33.5 vs. 18, *p* < 0.001) periods when compared to the pre-pandemic (Table 1). In addition, the proportion of household income with less than $20,000 per year was higher during the pandemic (65.59% vs. 38.97%, *p* < 0.001) and post-pandemic (62.91% vs. 38.97%, *p* < 0.001) compared to pre-pandemic (Table 1). On the other hand, the proportion of females was higher than that of males in the pre-pandemic (63.72% vs. 36.28%), and this gap widened in the pandemic (82.74% vs. 17.26%) and post-pandemic (84.86% vs. 15.14%) (Table 1).

**Table 1:** Distribution of HPV uptake and demographic characteristics in the pre-pandemic (2016–2019), during the pandemic (2020–2021), and post-pandemic (2022–2023) periods in El Paso, Texas

Factor	Overall	Before 2020	2020 and 2021	2022 and 2023	*p*-value
Total number of encounters	3709	1796	592	1321	
HPV Initiation					< 0.001
No	678 (18.28%)	181 (10.08%)	180 (30.41%)	317 (24.00%)	
Yes	3031 (81.72%)	1615 (89.92%)	412 (69.59%)	1004 (76.00%)	
Age, mean (SD)	25.68 (10.66)	17.96 (5.97) (n = 1680)	30.35 (10.25) (n = 581)	33.51 (8.37) (n = 1311)	< 0.001
Parent's level of education					< 0.001
None	111 (3.49%)	99 (7.81%)	11 (1.87%)	1 (0.08%)	
Till 10th	599 (18.85%)	114 (9.00%)	136 (23.09%)	349 (26.42%)	
Till 12th	1008 (31.73%)	342 (26.99%)	206 (34.97%)	460 (34.82%)	
Above 12th	1459 (45.92%)	712 (56.20%)	236 (40.07%)	511 (38.68%)	
Parent's household income					< 0.001
$0—$20,000 per year	1916 (51.76%)	698 (38.97%)	387 (65.59%)	831 (62.91%)	
$20,000 + per year	780 (21.07%)	304 (16.97%)	125 (21.19%)	351 (26.57%)	
Don't know/Refuse	1006 (27.17%)	789 (44.05%)	78 (13.22%)	139 (10.52%)	
Gender					< 0.001
Female	2704 (74.51%)	1094 (63.72%)	489 (82.74%)	1121 (84.86%)	
Male	925 (25.49%)	623 (36.28%)	102 (17.26%)	200 (15.14%)	

*Abbreviations*: *SD* Standard Deviation

### The COVID-19 pandemic affected HPV vaccine uptake

The total number of encounters with the HPV vaccine for the three periods was 3709. Among these encounters, the number of individuals who obtained at least one dose of the vaccine was 3031, or 81.72% of the total number of encounters. We observed a decrease in the total number of encounters for HPV vaccine during the pandemic (*N* = 592) and post-pandemic (*N* = 1321) periods compared to the pre-pandemic period (*N* = 1796). Similarly, there was a decrease in vaccine initiation rates during the pandemic (69.59%), which improved slightly in the post-pandemic period (76%), though they did not return to pre-pandemic levels (89.92%). These statistics are provided in Table 1.

**Fig. 1:**
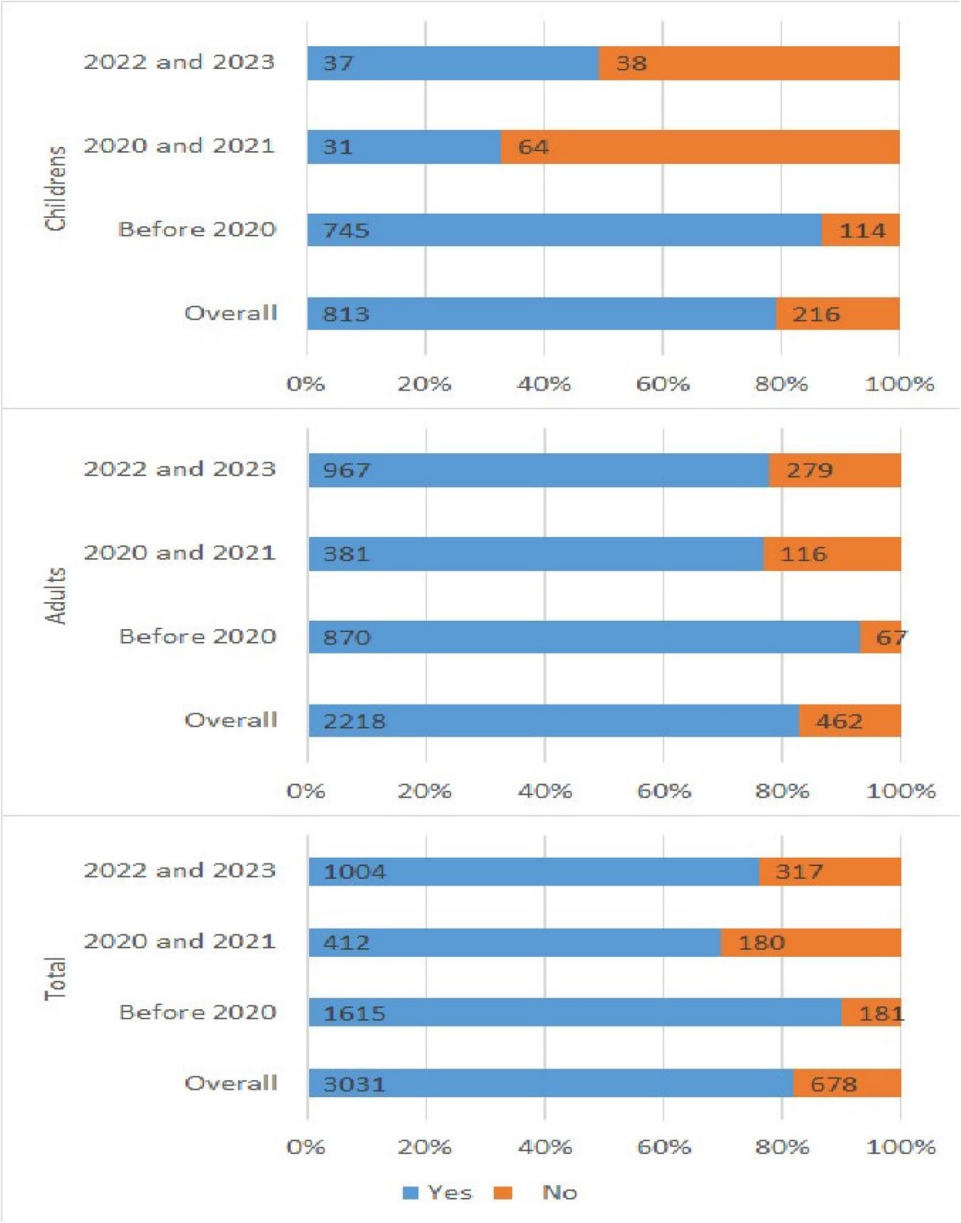
HPV initiation rate among encounters for the HPV vaccination for children and adults in the pre-pandemic (2016–2019), during the pandemic (2020–2021), and post-pandemic (2022–2023) periods in El Paso, Texas

Among encounters for the HPV vaccine among children, a significant reduction was observed in the uptake of the HPV vaccine during the pandemic (32.63% vs. 86.73%) and post-pandemic years (49.33% vs. 86.73%) compared to pre-pandemic. A similar pattern was also observed for adults in which those who received at least one dose of the HPV vaccine was 92.85% during pre-pandemic and reduced significantly to 76.66% and 77.61% during the pandemic and post-pandemic periods, respectively (Fig. 1).

### Covariates of HPV vaccine uptake

Unadjusted analysis indicated that the likelihood of obtaining the vaccine decreased significantly during the pandemic (OR: 0.257, 95% CI:[0.203, 0.324], *p* < 0.001) and post-pandemic (OR: 0.355, 95% CI:[0.291, 0.433], *p* < 0.001) as compared to pre-pandemic (Table 2). After adjusting for age, parent's level of education, household income, and gender, the odds of initiating the HPV vaccine series were even lower during the pandemic (OR: 0.091, 95% CI: [0.065, 0.129], *p* < 0.001) and post-pandemic (OR: 0.109, 95% CI:[0.078, 0.153), *p* < 0.001) compared to pre-pandemic period. We did not observe any association between participants' level of education (for children, parental level of education was collected), household income, and gender with the odds of initiating the vaccine, except for age (OR = 1.035, 95% CI: [1.023, 1.046], *p* < 0.001). These findings are presented in Table 2.

**Table 2:** Unadjusted and adjusted association of HPV initiation rate in the pre-pandemic (2016–2019), during the pandemic (2020–2021), and post-pandemic (2022–2023) periods in El Paso, Texas

Factor	OR (95% CI)	*p*-value
**Unadjusted**		
Period		
Before 2020	1 (reference)	
2020 and 2021	0.257 (0.203, 0.324)	< 0.001
2022 and 2023	0.355 (0.291, 0.433)	< 0.001
**Adjusted**		
Period		
Before 2020	1 (reference)	
2020 and 2021	0.091 (0.065, 0.129)	< 0.001
2022 and 2023	0.109 (0.078, 0.153)	< 0.001
Age, mean (SD)	1.035 (1.023, 1.046)	< 0.001
Parent's level of education		
None	1 (reference)	
Till 10th	0.467 (0.178, 1.222)	0.121
Till 12th	0.455 (0.176, 1.177)	0.104
Above 12th	0.504 (0.196, 1.298)	0.156
Parent's household income		
$0—$20,000 per year	1 (reference)	
$20,001 + per year	0.974 (0.764, 1.241)	0.829
Don't know/Refuse	0.779 (0.591, 1.026)	0.075
Gender		
Female	1 (reference)	
Male	0.905 (0.703, 1.166)	0.439

*Abbreviations*: *OR* Odds Ratio, *CI* Confidence Interval, *SD* Standard Deviation

## Discussion

Our findings reveal a substantial decrease in HPV vaccine uptake during the COVID-19 pandemic, with a persistent pattern observed post-pandemic. Similarly, there was a significant decline in the number of encounters with HPV vaccine outreach during the pandemic. There is concern about the substantially reduced proportion of those initiating HPV vaccinations per encounter, especially with the change in the age recommendations for HPV vaccination in 2019 to include adults aged 27 to 45 [26]. While we acknowledge that adults aged 27 to 45 were not eligible for the HPV vaccine in the pre-pandemic period due to the release of updated screening guidelines in 2019, our primary focus in this study was to analyze the overall HPV vaccination initiation rates during the pandemic period (2020–2021) and the post-pandemic period (2022–2023). Our data revealed a similar decline pattern separately for children and adults, prompting us to aggregate them. Recognizing that other variables such as age, education, gender, and income may influence the effect of HPV vaccination across the periods, we performed an adjusted analysis and presented the results in Table 2. These results demonstrated that even after accounting for age, education, income, and gender, HPV vaccination rates continued to decline during the pandemic and post-pandemic periods compared to the pre-pandemic period.

The COVID pandemic impacted routine care and, by extension, vaccine uptake. The World Health Organization (WHO) reported a 70% reduction in childhood vaccination rates at the beginning of the pandemic, with some countries reporting a 90% reduction rate [2]. A study conducted at the Children's Hospital of Los Angeles reported that Hispanic and African American groups had increased hesitancy towards childhood vaccinations due to the pandemic [16]. A systematic review of vaccine coverage across the globe showed that overall childhood vaccination rates decreased throughout 2020, resulting in a fourfold increase in polio outbreaks in polio-endemic countries [19]. The number of flu vaccines administered from January 2020 to August 2021 decreased, and researchers discovered that fear of exposure to COVID-19 was a leading factor [9]. A survey conducted by the CDC during the pandemic showed that half of the women surveyed had not received their Tdap or influenza vaccine during pregnancy due to increased vaccination hesitancy, and rates were even lower among Hispanic and Latino women compared to White women [38].

*Similarly, multiple studies confirmed a decline in the HPV vaccination rate during the COVID-19 pandemic *[43–45]*. Alarmingly, there appear to be persistent low HPV vaccination rates among uninsured participants *[35, 44]*. These studies supported our findings that the COVID-19 pandemic impacted vaccine uptake rates, significantly affecting uninsured individuals.*

Our study showed lower rates of HPV vaccine uptake in the pandemic and post-pandemic periods compared to the pre-pandemic period. There has been long-standing evidence of gender discrepancy in the uptake of the HPV vaccine, with HPV vaccination rates (1.1%-31.7%) in males being very low compared to females (2.4%-94.4%) [23]. While this phenomenon is complex and likely not attributed to a single causation, Daley and colleagues postulated a theory that "HPV and its associated interventions become feminized" to explain how the history of development and approval of the HPV vaccine was initially shaped by a sole emphasis on women's health [7]. Several studies estimate that HPV incidence and prevalence in men are similar to those of women, although substantial variability may exist by location and population [11]. HPV-related cases of oropharyngeal cancers in otherwise healthy men were predicted to surpass the number of cervical cancer cases in the United States by the year 2020 if HPV vaccination rates were not improved [5]. This happened sooner than expected, with new cervical cancer cases dropping each year as oropharyngeal cancers rose, 82% of the latter occurring in men [46]. As the burden of oropharyngeal cancer (OPC) is projected to grow among males [8], coupled with the rapid decline in HPV vaccine encounters and uptake due to the pandemic, it is crucial to explore strategies to reverse this harmful trend and prevent future excess OPC burden. One of the strategies being considered is a gender-neutral HPV vaccination program [6, 12], though more studies are needed to validate the adoption of this approach.

Several possibilities may explain why HPV vaccine uptake in this evidence-based, culturally tailored, multi-component program has yet to return to baseline despite the continued replication of program components, i*ncluding outreach education and provision of no-cost vaccines. These possibilities include increased vaccine hesitancy( noted nationally) and changes in healthcare priorities, with acute care ( delayed during the pandemic) prioritized over preventive care like vaccines.* However, it is essential to keep a pulse on changes in vaccine hesitancy in the general population, which tends to be more robust against perceived newer vaccines. The WHO listed vaccine hesitancy among the top 10 threats to world health [30]. Historically, democracies have been associated with improved health outcomes due to higher wealth levels, increased public health outreach, and public institution accountability [14].

Research studies have shown that using social media as a source of vaccine information without any other trusted source was associated with vaccine hesitancy [1, 40, 47]. A meta-analysis conducted on vaccine hesitancy showed that there had been a distinct rise in the phenomenon since COVID-19, with a 293% increase in articles published concerning vaccine hesitancy from the pre-COVID to post-COVID period, emphasizing the gravity of this issue and the necessity for public health measures to combat this phenomenon [22]. Therefore, more studies are needed to explore the impact of social media misinformation on disrupting public health efforts to promote vaccine coverage in underserved Hispanic communities.

One of the limitations of this study is that we utilized a cross-sectional study design with its inherent limitations in establishing cause-and-effect relationships. The results of this study may not be generalizable to other ethnic groups as this study was conducted in a community on the US-Mexico border, and findings may differ in non-border-dwelling Hispanic communities. In addition, most of our study participants self-identified as individuals of Hispanic origin, much higher than many studies reporting effects on Hispanic populations. However, although this limits generalizability, it is also a strength as it provides much-needed information about health promotion interventions in this underrepresented group. Despite these limitations, our study has some unique strengths. Ours is the first community-based study to assess the impact of the COVID-19 pandemic on a multifaceted intervention on both HPV vaccine-eligible adults and children in a predominantly underserved Hispanic population on the US-Mexico border. Despite vaccination disruption caused by the pandemic, we were able to adapt program delivery protocols to incorporate telephone and virtual platforms such as Zoom and Webex to provide health education as well as vaccine vouchers for participants to receive their vaccines at their local Walgreens or Immunize El Paso office. The program also ran a social media campaign that posted culturally tailored messaging and short health education videos targeted to parents of children/adolescents and young adults.

## Conclusion

Our findings suggest that the COVID-19 pandemic significantly impacted HPV vaccine uptake in an underserved Hispanic community. As we move forward, public health officials must explore strategies to address vaccine hesitancy, adapt interventions to the community's evolving needs, and mitigate the impact of future outbreaks on routine preventive care and efforts to reduce health disparities in underserved communities.

## Data Availability

No datasets were generated or analysed during the current study.
